# The effects of vitamin B and D supplementations on autonomic functions and quality of life in children after vasovagal syncope

**DOI:** 10.3389/fped.2025.1553428

**Published:** 2025-03-11

**Authors:** Tetiana Kovalchuk, Oksana Boyarchuk, Nataliya Balatska

**Affiliations:** ^1^ Department of Pediatrics # 2, Ivan Horbachevsky Ternopil National Medical University of the Ministry of Health of Ukraine, Ternopil, Ukraine; ^2^ Department of Pediatrics and Pediatric Surgery, Ivan Horbachevsky Ternopil National Medical University of the Ministry of Health of Ukraine, Ternopil, Ukraine; ^3^ Pediatrics Department # 1, Bogomolets National Medical University, Kyiv, Ukraine

**Keywords:** vitamin D, vitamin B, heart rate variability, blood pressure variability, health-related quality of life, vasovagal syncope, children

## Abstract

**Aim of the study:**

The aim of the study was to assess the effectiveness of vitamin B6, B9, B12, and D3 supplements in reducing symptoms and the frequency of syncope, improving autonomic nervous system functions, and enhancing quality of life (QOL) in children following an episode of vasovagal syncope (VVS).

**Materials and methods:**

The study involved 68 adolescents with VVS who consistently took vitamin B and D supplements and returned for the examination after 3 months. The effectiveness of the therapy was assessed using the CSSS (Calgary Syncope Seizure Score) and MCSSS (Modified Calgary Syncope Seizure Score), serum vitamin profiles (measured using the ELISA method), heart rate variability, blood pressure variability, and the PedsQL™ (Pediatric Quality of Life Inventory™) 4.0 Generic Core Scales and PedsQL™ 2.0 Family Impact Module surveys.

**Results:**

The study demonstrated that 3 months of vitamin supplementation were associated with a significant reduction in the frequency of symptoms and syncope episodes (*p* < 0.05). A marked decrease in serum homocysteine levels was observed, from 13.8 (9.9–17.9) µmol/L to 8.5 (7.6–10.8) µmol/L (*p* < 0.001). Vitamin supplementation also resulted in improved heart rate variability, evidenced by a significant increase in the SDANN index (*p* = 0.03) and reductions in TP (*p* = 0.002), LF (*p* = 0.004), and LF/HF (*p* = 0.01), indicating a decrease in sympathotonic influences on the cardiovascular system. Additionally, improved cardiac autonomic function in children with VVS during therapy was reflected by a higher prevalence of dipper profiles for systolic (*p* = 0.008) and diastolic (*p* < 0.001) blood pressure. During the 3-month therapy, the QOL in children showed improvements in physical, emotional, and school functioning (*p* < 0.05). In parents of children with a history of VVS, there were enhancements in physical, emotional, social, and cognitive functioning, as well as in communication and a reduction in worry levels. Among family members, daily activities and family relationships also improved (*p* < 0.05).

**Conclusion:**

The use of vitamin B6, B9, B12, and D3 supplements in therapeutic and preventive doses over 3 months in patients with a history of VVS is associated with a reduction in symptoms and syncope frequency, a decrease in serum homocysteine levels, a reduction in autonomic dysregulation, and an improvement in the QOL for children and their families.

## Introduction

Over the past two decades, significant progress has been made in understanding various aspects of the diagnosis and treatment of syncope. A substantial part of this progress lies in the establishment of clear criteria necessary for diagnosing syncope (1). The diversity of pathophysiological mechanisms and clinical patterns of transient loss of consciousness, as well as the irrational use of numerous costly laboratory and instrumental investigations, significantly impact the final clinical-diagnostic and financial-economic outcomes (2–4). Current methods for managing non-cardiас syncope focus on educating children and their relatives about physical measures to counteract symptoms and prevent fainting. However, there is no clear consensus among pediatric specialists regarding the use of other pharmacological and non-pharmacological interventions (5–7). These facts highlight the importance of optimizing the diagnostic and therapeutic process for vasovagal syncope (VVS), which is the most common cause of transient loss of consciousness in childhood.

Recent scientific research suggests that vitamin B12 deficiency and hyperhomocysteinemia are involved in the pathogenesis of syncope (8–11). Additionally, the role of vitamin D deficiency in the development of autonomic dysfunction, disruptions in circadian blood pressure (BP) rhythms, and the severity of symptoms in children with VVS has been established (12–14). Although the causal relationships between folate cycle indicators, vitamin D, and syncope remain insufficiently studied, they provide a window of opportunity for exploring new pathogenetically grounded methods of treatment and prevention of fainting. Despite the high relevance of this issue, no studies have yet evaluated the effectiveness of vitamin D in patients with VVS (15). There are only isolated reports of the positive effects of cyanocobalamin in reducing VVS symptoms and recurrence rates in adults (16, 17), and no data exist regarding the effectiveness of B-group vitamin supplements in the pediatric population with VVS. Therefore, the aim of this study was to assess the effectiveness of B6, B9, B12, and D3 vitamin supplements in reducing symptoms and syncope frequency, improving autonomic nervous system functions, and enhancing health-related quality of life (HRQOL) in children following an episode of VVS.

## Materials and methods

### General characteristics of the examined group of children

Inclusion criteria: syncope triggered by pain, fear, or prolonged standing and associated with a typical progressive prodromal period (pallor, sweating, and/or nausea); at least one syncope episode within the past month; normal response to active orthostatic testing; absence of structural heart disease and electrocardiography findings suggestive of arrhythmogenic syncope; no signs of epileptiform activity on electroencephalogram; absence of any other evident etiology of syncope.

Exclusion criteria: use of vitamin B and/or D supplements within the past year; seasonal period from May to August; presence of overweight, obesity, hypertension, anemia, malabsorption syndrome, chronic kidney diseases, other acute or exacerbation of chronic illnesses; inability to read and write in Ukrainian.

Inclusion criteria for parents or guardians' participation in surveys: ability to read and write in Ukrainian; being the primary caregiver of the child for at least the past year.

Ethical approval for this clinical study was obtained from the Ivan Horbachevsky Ternopil National Medical University of the Ministry of Health of Ukraine. All participants signed written informed consent before the study.

### Determination of serum levels of vitamins B6, B9, B12, D, and homocysteine

Each participant had 5 ml of blood drawn from the cubital vein between 8:00 and 11:00 a.m. in a fasting state. The collected blood was centrifuged within 30 min at a speed of 3,000 rpm for 3–5 min. Serum samples were frozen at −80°C until analysis. The concentrations of pyridoxine, folates, cyanocobalamin, 25-hydroxyvitamin D [25(OH)D], and homocysteine were measured in the serum using the quantitative enzyme-linked immunosorbent assay (ELISA) method. The results of the vitamin status evaluation and homocysteine profile in the examined children are presented in Table 1.

**Table 1:** Status of serum pyridoxine, folic acid, cyanocobalamin, 25(OH)D, and homocysteine in children with VVS before treatment.

Parameter status	VVS *n* (%)	Measurement range
Pyridoxine		
Optimal	39 (57.4)	>7.41 μg/L (18)
Suboptimal	21 (30.9)	7.41–4.94 μg/L (18)
Deficiency	8 (11.7)	<4.94 μg/L (18)
Folates		
Optimal	23 (33.8)	>4.5 μg/L (19)
Suboptimal	35 (51.5)	3–4.5 μg/L (19)
Deficiency	10 (14.7)	<3 μg/L (19)
Cyanocobalamin		
Optimal	31 (45.6)	>300 ng/L (19, 20)
Suboptimal	30 (44.1)	200–300 ng/L (19, 20)
Deficiency	7 (10.3)	<200 ng/L (19, 20)
25(OH)D		
Optimal	6 (8.8)	30–50 ng/L (21)
Suboptimal	23 (33.8)	20–30 ng/L (21)
Deficiency	39 (57.4)	20 ng/L (21)
Homocysteine		
Optimal	31 (45.6)	<95th percentile (22)
Hyperhomocysteinemia	37 (54.4)	≥95 percentile (22)

### Method of pharmacological correction of vitamins deficiency and insufficiency

All children involved in the study with a diagnosis of syncope were prescribed a vitamin supplement containing pyridoxine, folic acid, and cyanocobalamin, depending on their baseline serum levels. Pyridoxine (2 mg), folic acid (0.4 mg), and cyanocobalamin (0.006 mg) were administered daily to children with deficiency and every other day for those with optimal or suboptimal vitamin status for a period of 3 months. The choice of dosage for vitamins B6, B9, and B12 was based on age-related daily vitamin requirements and recommendations for the treatment of hypovitaminosis (18, 19, 23). The preventive dose of cholecalciferol for children with optimal or suboptimal vitamin D status was 1,000 IU. In the case of vitamin D deficiency, the therapeutic dose of cholecalciferol was 4,000 IU for 3 months (21). Thus, the dosage of vitamins B and D was based on the assessment of serum vitamin levels (deficient, insufficient, and optimal status) and the child's age. The weight criterion was not considered in vitamin dosing, as all children with overweight and obesity were excluded from the study.

### Methods of assessing therapy effectiveness

The effectiveness of vitamin supplements in the treatment and prevention of recurrent syncope episodes was assessed through careful collection of complaints and medical history using the CSSS (Calgary Syncope Seizure Score) and MCSSS (Modified Calgary Syncope Seizure Score) scales before treatment, at 3 and 12 months. Vitamin levels in the blood were monitored by measuring serum profiles of pyridoxine, folates, cyanocobalamin, homocysteine, and 25(OH)D using ELISA method after 3 months of treatment. The functioning of the autonomic nervous system was evaluated using heart rate variability (HRV) indicators (24-h Holter monitoring) and the circadian type of the daily BP profile (24-h ambulatory BP monitoring) before treatment and after 3 months.

The 24-h Holter monitoring was conducted during 24 h of usual daily activity in stationary or outpatient settings. All intensive physical activities were excluded on that day. The analysis of the main 24-h Holter monitoring parameters was performed using software after manually removing all artifacts from the recording. For the analysis of HRV, 3 time-domain (SDАNN, standard deviation of the averages of NN intervals; RMSSD, root mean square of successive differences; pNN50, percentage of NN intervals differing by more than 50 ms) and 5 frequency-domain (TP, total power; VLF, very low-frequency power; LF, low-frequency power; HF, high-frequency power; LF/HF, ratio of low-frequency to high-frequency power) parameters of 24-h Holter monitoring were used.

24-h ambulatory BP monitoring was performed using an oscillometric monitor with a properly sized cuff over a 24-h period during normal daily activities. BP was measured on the “non-dominant” arm, but in cases of asymmetry greater than 10 mm Hg, it was measured on the arm with the higher BP value. Automatic measurements were taken every 15 min during the day and every 30 min at night. Four categories of nocturnal BP reduction were identified: dippers (24-h index of 10%–22%), non-dippers (24-h index of 0%–10%), over-dippers (24-h index above 22%), and night-peakers (24-h index below 0).

To assess the psychosocial effects of the proposed therapy, children and their parents/caregivers were surveyed using the PedsQL™ (Pediatric Quality of Life Inventory™) 4.0 Generic Core Scales and PedsQL™ 2.0 Family Impact Module before and 3 months after vitamin supplements. These questionnaires were used in the study only after obtaining official permission from the developer, J.W. Varni, through the signing of a user agreement with MAPI Research Trust (Lyon, France).

### Statistical methods used in the study

The data analysis was performed using the SPSS 12.0 software package for Windows. Hypothesis testing was conducted using both parametric and non-parametric tests. Quantitative measurements with normal data distribution were presented as M ± SD (Mean ± Standard Deviation). Quantitative variables that deviated from normal distribution were expressed as medians (IQR, interquartile range). The results of qualitative measurements were presented as counts (*n*) and percentages (%). A comparative analysis of two dependent samples with normal distribution was performed using the Student's *t*-test, while for non-normal distributions, the Wilcoxon signed-rank test was used. The *χ*²-Pearson test was applied to compare the frequency of effects between two groups. A significance level of *p* < 0.05 indicated statistically significant differences.

The validation of the HRQOL questionnaires consisted of evaluations for format and data analysis (relevance criterion), reliability (internal consistency criterion), and validity (construct and criterion-related validity). To assess the relevance criterion of the questionnaires, the percentage of missing values for each item, the distribution of responses to each question within the survey, as well as the percentage of responses with the maximum possible score (“ceiling” effect) and the minimum possible score (“floor” effect) were determined. The reliability of the questionnaires was assessed by evaluating internal consistency using Cronbach's α coefficient. Construct validity was evaluated using factor analysis. The agreement between the child's self-report and the parent proxy report within a single questionnaire was studied using the intraclass correlation coefficient and Cohen's d effect size.

## Results

The study included 83 children with VVS, aged 12–17 years, consisting of 42 girls and 41 boys. The average age of the participants was 15.0 ± 2.1 years, with the average age at the onset of the first syncopal episode being 13.4 ± 2.8 years. At the time of enrollment, the average number of prior syncopal episodes recorded in the children's medical histories was 4.1 ± 1.6. All 83 children had their baseline vitamin levels measured, and a follow-up examination 3 months after the start of treatment was recommended. However, only 68 patients (35 girls and 33 boys) consistently took the vitamin supplements at the prescribed doses and returned for the examination after 3 months. The attrition rate of 18%, resulting in the loss of 15 participants, is considered acceptable.

During 3 months of therapy with the combined vitamin supplement, only 4 (5.9%) children experienced a repeat episode of uncomplicated syncope. On therapy, the VVS group showed a significant decrease in the total CSSS score [2 (0–3) points to 0 (0–0) points; *p* = 0.001] and an increase in the MCSS score (−2 [(−4)-(−2)] points to 0 (0–0) points; *p* < 0.001). Over the course of a year from the start of therapy, repeat episodes of syncope were recorded in 8 (11.8%) of cases. Compared to baseline data, significant improvements were still observed: CSSS score remained significantly low [0 (0–1) points; *p* = 0.01], while the MCSS score remained significantly high [0 (0–0) points; *p* < 0.001].

The control of the vitamin content in the blood after 3 months of treatment showed an increase in the serum concentrations of pyridoxine [from 8.1 (5.8–11.8) µg/L to 13.9 (9.2–16.3) µg/L; *p* < 0.001], folic acid [from 3.7 (3.0–4.7) µg/L to 6.6 (5.7–8.2) µg/L; *p* < 0.001], cyanocobalamin [from 293.3 (234.8–323.2) ng/L to 411.1 (358.5–511.4) ng/L; *p* < 0.001], and 25(OH)D [from 17.8 (13.9–23.6) ng/ml to 33.5 (29.4–38.2) ng/ml; *p* < 0.001]. These results were associated with a significant decrease in the serum homocysteine level [from 13.8 (9.9–17.9) µmol/L to 8.5 (7.6–10.8) µmol/L; *p* < 0.001].

The changes in HRV indicators after 3 months of treatment in children with a history of syncope were characterized by an increase in SDANN (standard deviation of the averages of NN intervals), and a decrease in TP (total power), LF (low-frequency power), and LF/HF (ratio of low-frequency to high-frequency power), indicating a reduction in sympathetic-adrenal influences on heart rate (Table 2).

**Table 2:** HRV before and after treatment.

Indicator	Before treatment median (IQR)	3 months after treatment median (IQR)	*р*
SDАNN, ms	207 (148–353)	348 (302–425)	**0** **.** **034**
RMSSD, ms	236 (175–433)	377 (314–412)	0.097
pNN50, %	33 (21–40)	32 (27–39)	0.431
TP, ms^2^	7,500 (4,872–11,871)	5,711 (4,892–6,625)	**0**.**002**
VLF, ms^2^	3,022 (2,196–4,745)	3,440 (2,078–4,826)	0.400
LF, ms^2^	2,311 (1,178–3,873)	1,910 (1,268–2,189)	**0**.**004**
HF, ms^2^	1,267 (813–2,946)	1,570 (1,157–2,108)	0.555
LF/HF	1.4 (1.0–2.3)	1.2 (1.1–1.2)	**0**.**010**

SDАNN, standard deviation of the averages of NN intervals; RMSSD, root mean square of successive differences; pNN50, percentage of NN intervals differing by more than 50 ms; TP, total power; VLF, very low-frequency power; LF, low-frequency power; HF, high-frequency power; LF/HF, ratio of low-frequency to high-frequency power; IQR, interquartile range.

Bold values are statistically significant data.

After 3 months of treatment, changes in the circadian type of the daily BP profile were accompanied by a significant increase in the dipper pattern for systolic blood pressure (SBP) and diastolic blood pressure (DBP, Table 3).

**Table 3:** Nocturnal blood pressure dipping in VVS patients before and after treatment.

BP	Dipping pattern	Before treatment *n* (%)	3 months after treatment *n* (%)	*χ* ^2^	*р*
SBP	Dippers	36 (53.0)	51 (75.0)	**7** **.** **178**	**0**.**008**
	Non-dippers	28 (41.2)	15 (22.0)	**5**.**747**	**0**.**017**
	Over-dippers	2 (2.9)	1 (1.5)	0.341	0.560
	Night-peakers	2 (2.9)	1 (1.5)	0.341	0.560
DBP	Dippers	40 (58.8)	58 (85.3)	**11**.**832**	**<0**.**001**
	Non-dippers	5 (7.3)	3 (4.4)	0.531	0.467
	Over-dippers	22 (32.4)	6 (8.8)	**11**.**513**	**<0**.**001**
	Night-peakers	1 (1.5)	1 (1.5)	0.000	1.000

BP, blood pressure; SBP, systolic blood pressure; DBP, diastolic blood pressure.

Bold values are statistically significant data.

The psychometric analysis of the PedsQL™ 4.0 Generic Core Scales revealed that the missing data rate for self-reports from children and proxy-reports from parents was 0.7% and 1.4%, respectively, with “ceiling” and “floor” effects not exceeding 15% in the group of children with VVS. The Cronbach's alpha correlation coefficient was 0.96 for the child's self-report and 0.94 for the parent's proxy-report, indicating a high degree of internal consistency between the child and parent versions of the PedsQL™ 4.0 Generic Core Scales. All scales of the PedsQL™ 4.0 Generic Core Scales showed strong (≥0.70) correlations with the overall QOL factor, confirming their construct validity. The obtained intraclass correlation coefficients (r) demonstrated good agreement between the child's self-report and the parent's proxy-report within the PedsQL™ 4.0 Generic Core Scales: physical functioning—0.76; emotional functioning—0.75; social functioning—0.72; school functioning—0.72; physical health summary score—0.76; psychosocial health summary score—0.76; total scale score—0.81 (*p* < 0.001). Cohen's d effect size ≤0.20 within the PedsQL™ 4.0 Generic Core Scales further indicates no statistically significant difference between children's self-reports and parents' proxy-reports in the VVS group: physical functioning—0.19; emotional functioning—0.03; social functioning—0.16; school functioning—0.12; physical health summary score—0.19; psychosocial health summary score—0.08; total scale score—0.14.

According to the results of the PedsQL™ 4.0 Generic Core Scales survey, 3 months after the use of vitamin B6, B9, B12, and D3 supplements, improvements were observed in physical, emotional, and school functioning, along with an increase in the total QOL score in children who had recently experienced VVS (Table 4).

**Table 4:** QOL indicators in children with VVS before and after treatment.

PedsQL™ 4.0 generic core scales	Before treatment M ± SD	3 months after treatment M ± SD	*р*
Child self-report			
Physical functioning	73.7 ± 16.2	81.1 ± 10.1	**<0**.**001**
Emotional functioning	68.5 ± 16.8	72.1 ± 14.5	**0**.**012**
Social functioning	79.1 ± 18.0	83.1 ± 10.9	0.051
School functioning	64.8 ± 17.5	82.8 ± 7.9	**<0**.**001**
Physical health summary score	73.7 ± 16.2	81.1 ± 10.1	**<0**.**001**
Psychosocial health summary score	70.9 ± 13.7	79.3 ± 8.0	**<0**.**001**
Total scale score	71.8 ± 13.1	79.9 ± 7.9	**<0**.**001**
Parent proxy-report			
Physical functioning	67.3 ± 20.2	80.1 ± 8.9	**<0**.**001**
Emotional functioning	68.2 ± 16.3	71.2 ± 11.5	0.225
Social functioning	76.9 ± 19.5	81.3 ± 9.0	0.098
School functioning	60.6 ± 18.8	81.8 ± 7.1	**<0**.**001**
Physical health summary score	67.3 ± 20.2	80.1 ± 8.9	**<0**.**001**
Psychosocial health summary score	68.6 ± 15.2	78.1 ± 6.5	**<0**.**001**
Total scale score	68.2 ± 14.8	78.8 ± 6.7	**<0**.**001**

M ± SD, mean ± standard deviation.

Bold values are statistically significant data.

The psychometric assessment of the PedsQL™ 2.0 Family Impact Module revealed that the percentage of missing values for each item did not exceed 2.4%. The “ceiling” and “floor” effects within the questionnaire did not exceed 15% for each scale. These results met the established measurement standards and indicated that the PedsQL™ 2.0 Family Impact Module was appropriate for surveying family members of children with syncope. The Cronbach's α coefficient was 0.93, confirming the reliability of the PedsQL™ 2.0 Family Impact Module in assessing the QOL of family members in VVS group. All scales of the PedsQL™ 2.0 Family Impact Module showed strong (≥0.70) correlations with the HRQOL factor, except for the communication and worry scales, which proved moderate correlations (0.67 and 0.57).

It has been demonstrated that vitamin supplementations over 3 months in children with recent VVS had a positive impact on the parents' QOL, family functioning, and the overall family QOL (Table 5).

**Table 5:** Dynamics of HRQOL among family members of children with VVS during proposed therapy.

PedsQL™ 2.0 family impact module	Before treatment M ± SD	3 months after treatment M ± SD	*р*
Physical functioning	59.2 ± 17.8	73.8 ± 9.6	**<0** **.** **001**
Emotional functioning	59.3 ± 20.5	72.6 ± 12.8	**<0**.**001**
Social functioning	70.4 ± 20.3	77.6 ± 14.7	**<0**.**001**
Cognitive functioning	70.7 ± 20.0	78.6 ± 13.7	**<0**.**001**
Communication	63.0 ± 24.7	77.0 ± 14.7	**<0**.**001**
Worry	43.8 ± 19.9	69.3 ± 11.6	**<0**.**001**
Daily activities	52.0 ± 20.8	70.6 ± 13.8	**<0**.**001**
Family relationships	73.8 ± 19.5	81.8 ± 12.4	**0**.**001**
Parent HRQOL summary score	64.3 ± 16.7	75.4 ± 10.0	**<0**.**001**
Family summary score	65.6 ± 16.9	77.6 ± 10.9	**<0**.**001**
Total impact score	61.7 ± 14.0	75.2 ± 8.4	**<0**.**001**

HRQOL, health-related quality of life; M ± SD, mean ± standard deviation.

Bold values are statistically significant data.

## Discussion

Despite significant advances in understanding the mechanisms underlying VVS in recent years, including the abnormal Bezold-Jarisch reflex, autonomic nervous system dysfunction, neurohumoral, and hereditary factors, the pathogenesis of this condition remains poorly understood, debated, and in need of further investigation into other potential etiopathogenic factors (1, 3, 6). Recent scientific studies suggest a likely indirect, and in some cases, direct role of vitamin B and D deficiencies and hyperhomocysteinemia in the pathogenesis of syncope (8, 10, 13).

It has been proven that vitamin B12 deficiency may influence the development of the pathological Bezold-Jarisch reflex through impaired myelination, leading to slowed nerve conduction and increased serum norepinephrine levels (8, 20). As is well known, this catecholamine regulates the adaptive and compensatory functions of the cardiovascular system by increasing myocardial contractility, heart rate, cardiac output, and BP (24). Some studies demonstrate the impact of hyperhomocysteinemia on the autonomic nervous system via activation of the renin-angiotensin-aldosterone system and the development of sympathicotonia (25). Furthermore, hyperhomocysteinemia is associated with reduced HRV and increased BP variability (26).

The mechanisms by which vitamin D deficiency influences the development of VVS remain poorly understood and controversial. It has been established that excessive contractions of the left ventricle trigger the activation of cardiac C-fibers during the Bezold-Jarisch reflex, while vitamin D deficiency is associated with impaired cardiac autonomic function due to suppressed vagal balance (12, 13). Furthermore, vitamin D is involved in the proliferation and development of smooth muscle and endothelial cells, contributing to vascular elasticity (27). Its deficiency may lead to syncope through reduced peripheral vascular resistance during the pathological Bezold-Jarisch reflex in response to a trigger (15, 17). The likelihood of this mechanism is supported by observations from Usalp S. et al., who reported significantly lower vitamin D levels in tilt-positive patients compared to tilt-negative individuals (28). Another potential pathophysiological link in syncope could be disrupted neuronal conduction of the baroreflex mechanism. Vitamin D, present in both the central and peripheral nervous systems, plays a critical role in maintaining the neurotrophic and neuroprotective effects of growth factors involved in neurotransmitter conduction and nerve cell growth (29).

This study revealed a significant prevalence of vitamin insufficiencies and deficiencies, particularly for vitamins B6, B9, B12, and D. These deficiencies were linked to a high occurrence of hyperhomocysteinemia in children with VVS. The findings highlight the necessity of vitamin supplementation, with subsequent assessment of its effectiveness in alleviating symptoms, reducing syncope episodes, improving autonomic nervous system function, and enhancing HRQOL.

Despite the high relevance of this issue, studies on the efficacy of vitamin D in patients with VVS have not yet been conducted (15). There are only isolated reports on the positive effects of cyanocobalamin in reducing VVS symptoms and decreasing recurrence rates in adults (16, 17), while data on the efficacy of B vitamins in children with VVS are completely lacking. At the same time, it has been proven that B vitamin supplementation can effectively alleviate symptoms of autonomic dysfunction by reducing serum homocysteine levels (30). Considering the fact that vitamin D may regulate the expression of genes involved in homocysteine metabolism enzymes, an increasing number of studies support the efficacy of vitamin D3 supplementation in treating hyperhomocysteinemia (31). In our study, the effectiveness of oral supplementation with vitamins B6, B9, B12, and D3 in the doses described above for 3 months was confirmed by a significant reduction in serum homocysteine levels (*p* < 0.001).

Studies on the effectiveness of vitamin B and D supplementation in improving autonomic nervous system function are extremely limited and fail to shed light on the pathophysiological mechanisms of this interaction. Zhong et al. demonstrated the ability of B vitamin supplements to influence autonomic regulation of the cardiovascular system, as evidenced by a decrease in heart rate HR and an increase in HRV among healthy adults (32). Similarly, B vitamin supplementation in healthy individuals reduced the adverse effects of air pollution on cardiac autonomic dysfunction (33). Mann et al. showed that vitamin D supplementation improves cardiac autonomic tone in hemodialysis patients with 25(OH)D deficiency (34). Another study demonstrated that vitamin D and calcium supplementation in patients with vitamin D deficiency and insufficiency may modulate the sympathetic nervous system, leading to changes in BP and heart rate (35). Furthermore, it was showed that low-dose vitamin D intake enhances the sensitivity of the bradycardic component of the baroreflex in rats (36). It has been proven in another study that daily vitamin D intake leads to improvements in blood pressure levels and autonomic balance in individuals with overweight and obesity (37). These findings underscore the need for further research to better understand the pathophysiological mechanisms and the potential efficacy of vitamin supplementations in controlling cardiac autonomic dysfunction symptoms.

In our study, a 3-month course of vitamin supplementation was shown to increase HRV, as evidenced by a significant rise in the SDANN index, along with a decrease in TP, LF, and LF/HF (*p* < 0.01). These findings confirm a reduction in sympathotonic influences on the cardiovascular system. Another indication of improved cardiac autonomic function observed in our patients with VVS during therapy is the increased prevalence of dipper profiles for SBP and DBP (*p* < 0.01).

According to the World Health Organization Constitution, HRQOL serves as an additional tool for assessing health in a broader context, encompassing perceptions of health, psychosocial, mental well-being, and overall quality of life (38). The methodology for evaluating HRQOL in pediatric populations can help determine the burden of disease on both the child and their family members, as well as assess the effectiveness of therapeutic and preventive interventions (38–40). At the same time, studies addressing HRQOL in children with a history of VVS remain extremely limited. One such study identified lower HRQOL scores based on child self-reports compared to parent proxy-reports, influenced by the children's age and levels of parental stress (41). Shigeyasu et al. were the first to examine QOL in children with autonomic dysfunction, revealing low parameters of physical and emotional well-being, self-esteem, peer relationships, and school functioning (42). Numerous studies confirm the impact of autonomic nervous system imbalance on reduced HRQOL in children with autonomic disorders (43, 44).

In this study, the PedsQL™ 4.0 Generic Core Scales and PedsQL™ 2.0 Family Impact Module underwent psychometric evaluation, which confirmed their suitability, reliability, and validity when used with patients suffering from VVS. The results of the current research confirmed that the reduction in the frequency of symptoms and episodes of syncope, along with changes in autonomic nervous system functioning, were accompanied by improvements in the quality of life (QOL) for both children and their families during vitamin supplementation therapy. In children, there was an increase in physical, emotional, and school functioning (*p* < 0.05). In parents of children with a history of VVS, these changes were accompanied by improvements in physical, emotional, social, and cognitive functioning, communication, and a decrease in worry levels. Among family members, daily activities and family relationships also improved (*p* < 0.05).

This study has several limitations. The obtained results are based on a relatively small group of children and the absence of a healthy control group. It is also not definitively known what changes in HRV, BP variability, and HRQOL indicators occur 3 months after VVS in children who did not receive vitamin supplementations. This study also did not take into account the dietary habits of the children included in the research, which could have provided more insight into understanding the causes of vitamins insufficiency and deficiency in VVS. The effectiveness of treatment was monitored only after 3 months, so the long-term effectiveness of this approach at 6, 12, and more months remains unknown. The study presents results of correcting vitamin imbalances in pyridoxine, folates, cyanocobalamin, and 25(OH)D, whereas the impact of other vitamins on disease progression and treatment outcomes was not studied.

## Data Availability

The raw data supporting the conclusions of this article will be made available by the authors, without undue reservation.

## References

[1] Brignole M Moya A de Lange FJ Deharo JC Elliott PM Fanciulli A 2018 ESC guidelines for the diagnosis and management of syncope. Eur Heart J. (2018) 39(21):1883–948. 10.1093/eurheartj/ehy037 29562304

[2] Wang YY Du JB Jin HF. Differential diagnosis of vasovagal syncope and postural tachycardia syndrome in children. World J Pediatr. (2020) 16(6):549–52. 10.1007/s12519-019-00333-4 32020440

[3] Singhi P Saini AG. Syncope in pediatric practice. Indian J Pediatr. (2018) 85(8):636–40. 10.1007/s12098-017-2488-9 29119462

[4] Li Y Liu J Wang M Zhao H Liu X Hu J Predictive value of EGSYS score in the differential diagnosis of cardiac syncope and neurally mediated syncope in children. Front Cardiovasc Med. (2023) 10:1091778. 10.3389/fcvm.2023.1091778 37008325 PMC10063910

[5] Bagrul D Ece I Yılmaz A Atik F Kavurt AV. Midodrine treatment in children with recurrent vasovagal syncope. Cardiol Young. (2021) 31(5):817–21. 10.1017/S1047951120004746 33407967

[6] Cheng W Wang J Lin J. Biomarkers and hemodynamic parameters in the diagnosis and treatment of children with postural tachycardia syndrome and vasovagal syncope. Int J Environ Res Public Health. (2022) 19(12):6974. 10.3390/ijerph19126974 35742222 PMC9222341

[7] Wen C Wang S Zou R Wang Y Tan C Xu Y Duration of treatment with oral rehydration salts for vasovagal syncope in children and adolescents. Turk J Pediatr. (2023) 62(5):820–5. 10.24953/turkjped.2020.05.014 33108085

[8] Pektas A Koken R Koca HB. Serum vitamin B-12 in children presenting with vasovagal syncope. Asia Pac J Clin Nutr. (2018) 27(1):176–81. 10.6133/apjcn.022017.17 29222896

[9] Kovalchuk T Boyarchuk O. Serum pyridoxine, folate, cobalamin, and homocysteine levels in children presenting with vasovagal syncope. Cardiol Young. (2022) 32(5):762–8. 10.1017/S1047951121003036 34321136

[10] Li Y He B Li H Zhang Q Tang C Du J Plasma homocysteine level in children with postural tachycardia syndrome. Front Pediatr. (2018) 6:375. 10.3389/fped.2018.00375 30560108 PMC6287046

[11] Li Y Bai B Wang H Wu H Deng Y Shen C Plasma metabolomic profile in orthostatic intolerance children with high levels of plasma homocysteine. Ital J Pediatr. (2024) 50(1):52. 10.1186/s13052-024-01601-4 38486257 PMC10941598

[12] Zou R Wang S Cai H Li F Lin P Wang Y Vitamin D deficiency in children with vasovagal syncope is associated with impaired circadian rhythm of blood pressure. Front Neurosci. (2021) 15:712462. 10.3389/fnins.2021.712462 34456677 PMC8387869

[13] Zhang Q Sun Y Zhang C Qi J Du J. Vitamin D deficiency and vasovagal syncope in children and adolescents. Front Pediatr. (2021) 9:575923. 10.3389/fped.2021.575923 33732666 PMC7959715

[14] Kovalchuk T Boyarchuk O. Serum vitamin D levels in children and adolescents with vasovagal syncope, syncope due to orthostatic hypotension, and cardiac syncope. Turk Arch Pediatr. (2023) 58(1):42–8. 10.5152/TurkArchPediatr.2022.22141 36598210 PMC9885808

[15] Khalaji A Behnoush AH Tajdini M. Association between vitamin D deficiency and vasovagal syncope: a systematic review and meta-analysis. Clin Cardiol. (2023) 46(7):721–8. 10.1002/clc.24035 37226313 PMC10352974

[16] Hesselbrock RR Palileo EV Davenport ED. Vitamin B12 deficiency related syncope in a young military pilot. Aerosp Med Hum Perform. (2020) 91(9):746–8. 10.3357/AMHP.5650.2020 32867907

[17] Arora H Sastry BKS Narasimhan S Arora H. To study vitamin B12 deficiency and response to treatment in patients presenting with vasovagal syncope. J Cardiol. (2018) 2(5):000134. 10.23880/oajc-16000134

[18] Stover PJ Field MS. Vitamin B-6. Adv Nutr. (2015) 6(1):132–3. 10.3945/an.113.005207 25593152 PMC4288272

[19] Devalia V Hamilton MS Molloy AM. British Committee for standards in haematology. Guidelines for the diagnosis and treatment of cobalamin and folate disorders. Br J Haematol. (2014) 166(4):496–513. 10.1111/bjh.12959 24942828

[20] Öner T Guven B Tavli V Mese T Yilmazer MM Demirpence S. Postural orthostatic tachycardia syndrome (POTS) and vitamin B12 deficiency in adolescents. Pediatrics. (2014) 133(1):e138–42. 10.1542/peds.2012-3427 24366986

[21] Płudowski P Kos-Kudła B Walczak M Fal A Zozulińska-Ziółkiewicz D Sieroszewski P Guidelines for preventing and treating vitamin D deficiency: a 2023 update in Poland. Nutrients. (2023) 15(3):695. 10.3390/nu15030695 36771403 PMC9920487

[22] Huemer M Vonblon K Födinger M Krumpholz R Hubmann M Ulmer H Total homocysteine, folate, and cobalamin, and their relation to genetic polymorphisms, lifestyle and body mass index in healthy children and adolescents. Pediatr Res. (2006) 60(6):764–9. 10.1203/01.pdr.0000246099.39469.18 17065574

[23] Martini L Pecoraro L Salvottini C Piacentini G Atkinson R Pietrobelli A. Appropriate and inappropriate vitamin supplementation in children. J Nutr Sci. (2020) 9:e20. 10.1017/jns.2020.12 32577225 PMC7288613

[24] Lei LY Raj SR Sheldon RS. Pharmacological norepinephrine transporter inhibition for the prevention of vasovagal syncope in young and adult subjects: a systematic review and meta-analysis. Heart Rhythm. (2020) 17(7):1151–8. 10.1016/j.hrthm.2020.02.033 32151742 PMC7335357

[25] Liu L Wu Q Yan H Zheng X Zhou Q. Plasma homocysteine and autonomic nervous dysfunction: association and clinical relevance in OSAS. Dis Markers. (2020) 2020:4378505. 10.1155/2020/4378505 32695242 PMC7368224

[26] Lin BY Li P Wu XD Li H Zeng ZY. The relationship between homocysteine, blood pressure variability, and left ventricular hypertrophy in patients with essential hypertension: an observational study. Adv Ther. (2020) 37(1):381–9. 10.1007/s12325-019-01154-7 31755036

[27] Zhou W Wang W Yuan XJ Xiao CC Xing Y Ye SD The effects of RBP4 and vitamin D on the proliferation and migration of vascular smooth muscle cells via the JAK2/STAT3 signaling pathway. Oxid Med Cell Longev. (2022) 2022:3046777. 10.1155/2022/3046777 35082965 PMC8786468

[28] Usalp S Kemal H Yüksek Ü Yaman B Günsel A Edebal O Is there any link between vitamin D deficiency and vasovagal syncope? J Arrhythm. (2020) 36(2):371–6. 10.1002/joa3.12309 32256891 PMC7132194

[29] Gombash SE Lee PW Sawdai E Lovett-Racke AE. Vitamin D as a risk factor for multiple sclerosis: immunoregulatory or neuroprotective? Front Neurol. (2022) 13:796933. 10.3389/fneur.2022.796933 35651353 PMC9149265

[30] Sadeghvand S Barzegar M Shiva S Tarmahi V Hamed H Rahimi Khamaneh E The effects of vitamin B-Complex supplementation on serum homocysteine levels and migraine severity in children a randomized controlled trial. Iran J Child Neurol. (2023) 17(3):143–55. 10.22037/ijcn.v17i3.40053 37637782 PMC10448839

[31] Mazokopakis EE Papadomanolaki MG Papadakis JA. Associations of serum 25-hydroxyvitamin D with serum folate, cobalanin, and homocysteine concentrations and methylene tetrahydrofolate reductase (MTHFR) gene polymorphisms in healthy adults. Hormones (Athens). (2023) 22(3):491–9. 10.1007/s42000-023-00457-3 37328700

[32] Zhong J Trevisi L Urch B Lin X Speck M Coull BA B-vitamin supplementation mitigates effects of fine particles on cardiac autonomic dysfunction and inflammation: a pilot human intervention trial. Sci Rep. (2017) 7:45322. 10.1038/srep45322 28367952 PMC5377246

[33] Lim YH Park HY Yi SM Park E Lee BE Oh SY Influence of vitamin B deficiency on PM_2.5_-induced cardiac autonomic dysfunction. Eur J Prev Cardiol. (2020) 27(19):2296–8. 10.1177/2047487319888595 31813277

[34] Mann MC Exner DV Hemmelgarn BR Hanley DA Turin TC MacRae JM The VITAH trial-vitamin D supplementation and cardiac autonomic tone in patients with end-stage kidney disease on hemodialysis: a blinded, randomized controlled trial. Nutrients. (2016) 8(10):608. 10.3390/nu8100608 27690095 PMC5083996

[35] Tonnesen R Schwarz P Hovind P Jensen LT. Modulation of the sympathetic nervous system in youngsters by vitamin-D supplementation. Physiol Rep. (2018) 6(7):e13635. 10.14814/phy2.13635 29611325 PMC5880874

[36] Fioretti AC Dsouki NA do Vale B de Carvalho RP Dias DPM Venancio DP Vitamin D supplementation at different doses affects the vagal component of the baroreceptor reflex and the Bezold-Jarisch reflex in eutrophic rats. Front Physiol. (2022) 13:934625. 10.3389/fphys.2022.934625 35991180 PMC9389110

[37] Oigman W Neves MF. Effects of vitamin D supplementation on central hemodynamic parameters and autonomic nervous system in obese or overweight individuals. Arq Bras Cardiol. (2024) 121(5):e20230678. 10.36660/abc.20230678 38747749 PMC11098585

[38] Sitaresmi MN Indraswari BW Rozanti NM Sabilatuttaqiyya Z Wahab A. Health-related quality of life profile of Indonesian children and its determinants: a community-based study. BMC Pediatr. (2022) 22(1):103. 10.1186/s12887-022-03161-0 35193530 PMC8862365

[39] Kljajić M Maltese G Tarnow P Sand P Kölby L. Health-related quality of life of children treated for non-syndromic craniosynostosis. J Plast Surg Hand Surg. (2023) 57(1-6):408–14. 10.1080/2000656X.2022.2147532 36409664

[40] Kovalchuk T. Validation of the Ukrainian version of the PedsQLTM 4.0 generic core scales in children and adolescents with vasovagal syncope. Pediatr Pol. (2020) 95(2):112–20. 10.5114/polp.2020.97102

[41] Grimaldi Capitello T Fiorilli C Placidi S Vallone R Drago F Gentile S. What factors influence parents’ perception of the quality of life of children and adolescents with neurocardiogenic syncope? Health Qual Life Outcomes. (2016) 14:79. 10.1186/s12955-016-0476-9 27188269 PMC4869310

[42] Shigeyasu Y Okada A Fujii C Tanaka C Sugihara A Horiuchi M Quality of life and physical/psychosocial factors in children and adolescents with orthostatic intolerance. Biopsychosoc Med. (2023) 17(1):23. 10.1186/s13030-023-00278-1 37308984 PMC10258988

[43] Hertel A Black WR Malloy Walton L Martin J Jones J. Cardiovascular symptoms, dysautonomia, and quality of life in adult and pediatric patients with hypermobile Ehlers-Danlos syndrome: a brief review. Curr Cardiol Rev. (2024) 20(1):E240124226070. 10.2174/011573403X271096231203164216 38275067 PMC11071672

[44] Barbic F Minonzio M Cairo B Shiffer D Zamuner AR Cavalieri S Work ability assessment and its relationship with cardiovascular autonomic profile in postural orthostatic tachycardia syndrome. Int J Environ Res Public Health. (2020) 17(21):7836. 10.3390/ijerph17217836 33114659 PMC7662324

